# Nocturnal hypertension in kidney transplant recipients: epidemiology, pathophysiology, prognostic significance, and management

**DOI:** 10.1093/ckj/sfag114

**Published:** 2026-04-16

**Authors:** Areti Georgiou, Marieta Theodorakopoulou, Fotini Iatridi, Pantelis Sarafidis

**Affiliations:** First Department of Nephrology Aristotle University of Thessaloniki, Hippokration Hospital, Thessaloniki, Greece; First Department of Nephrology Aristotle University of Thessaloniki, Hippokration Hospital, Thessaloniki, Greece; First Department of Nephrology Aristotle University of Thessaloniki, Hippokration Hospital, Thessaloniki, Greece; First Department of Nephrology Aristotle University of Thessaloniki, Hippokration Hospital, Thessaloniki, Greece

**Keywords:** ABPM, dipping, hypertension, kidney transplantation, nocturnal hypertension

## Abstract

Kidney transplantation markedly improves survival and quality of life in patients with kidney failure; however, cardiovascular disease remains the leading cause of morbidity and mortality among kidney transplant recipients (KTRs). Hypertension after kidney transplantation is very common and is strongly associated with adverse cardiovascular as well as graft-related outcomes. Notably, hypertension in this population exhibits distinct features, including exceptionally high rates of masked hypertension; this is largely driven by the frequent occurrence of elevated nocturnal blood pressure (BP) and abnormal dipping patterns, which were observed in up to 95% of KTRs. Observational studies consistently demonstrate that higher nocturnal BP and abnormal dipping profiles are linked to accelerated graft function loss, cardiovascular events, and hypertension-mediated target-organ damage, frequently with stronger prognostic value than office or daytime BP measurements. In this review, we summarize the current evidence on circadian BP phenotypes and nocturnal hypertension in KTRs, focusing on epidemiology, underlying mechanisms, prognostic implications for cardiovascular and graft outcomes, and contemporary strategies for diagnosis and management.

## INTRODUCTION

Kidney transplantation is considered the optimal kidney replacement therapy for patients with kidney failure, as it is associated with longer survival, better quality of life, and lower costs compared to dialysis [1, 2]. Despite these benefits, kidney transplant recipients (KTRs) continue to experience markedly elevated cardiovascular risk relative to the general population, and cardiovascular disease remains the leading cause of long-term morbidity and mortality in this population [3, 4]. While kidney transplantation attenuates some risk factors associated with kidney failure and uremic environment, it introduces new mechanisms of cardiovascular injury (i.e. those related to side effects of immunosuppression or metabolic disturbances); the latter, combined with traditional and nontraditional pre-existing factors contribute to the substantial residual cardiovascular disease burden [5, 6].

Hypertension is among the most prevalent modifiable risk factors in KTRs. While blood pressure (BP) levels often improve early after transplantation [7–9] and are generally lower in KTRs than their dialysis counterparts [10], hypertension in the chronic phase affects ∼70%–95% of KTRs [11, 12], and is strongly associated with adverse cardiovascular and graft outcomes [13]. Importantly, hypertension in this population exhibits distinct features, such as the high rates of masked hypertension, mainly due to coexisting nocturnal hypertension and blunted or absent nighttime BP dipping [14].

In recent years, interest in the pathophysiology, proper diagnosis, and clinical management of nocturnal BP has increased substantially, driven by robust data linking nighttime hypertension to hypertension-mediated organ damage, cardiovascular events and kidney disease progression, often more strongly than daytime or office BP [15]. Accordingly, the importance of assessing nocturnal BP has been emphasized in recent hypertension recommendations [16, 17] and reinforced by recent position statements calling for greater clinical focus [15].

In this review, we summarize current evidence on circadian BP phenotypes and nocturnal hypertension in KTRs, carefully discussing epidemiology, underlying mechanisms, prognostic significance for cardiovascular and graft outcomes, and contemporary strategies for diagnosis and management.

### Search strategy

A detailed literature search of MEDLINE/PubMed database was performed to identify English language articles on humans published from database inception up to January 2026 that reported epidemiology data on nocturnal hypertension or dipping patterns in KTRs. Search terms used were ‘nocturnal’, ‘nighttime’, ‘blood pressure’, ‘hypertension’, ‘dipping’, ‘dipper’, ‘circadian’, ‘diurnal’, ‘kidney/renal transplant*’. Reference lists of identified articles were also evaluated for additional relevant articles and information. Original studies that examined either the prevalence of nocturnal hypertension and abnormal dipping patterns in KTRs or their associations with kidney and cardiovascular outcomes in this population were included.

### Circadian BP patterns and dipping profiles

**Figure 1:**
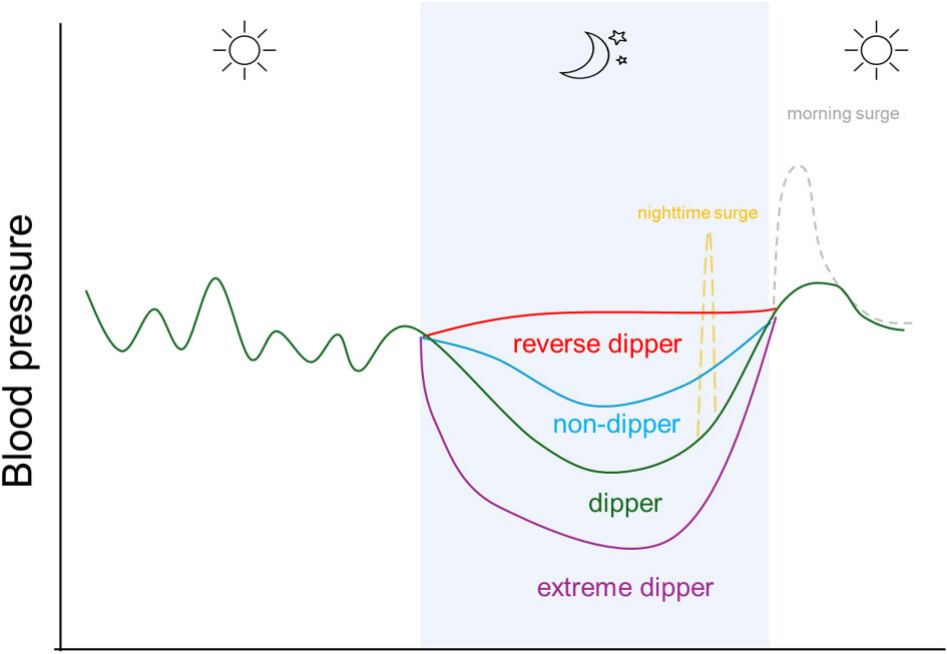
Circadian BP patterns and dipping phenotypes. Schematic representation of typical 24-h ambulatory BP profiles across different nocturnal dipping categories.

In healthy individuals, BP exhibits a specific circadian pattern, characterized by a 10%–20% decline during sleep (normal dipping) and an increase upon waking up (morning BP surge) (Fig. 1) [18]. This circadian rhythm of BP regulation is driven by both endogenous (i.e. central and peripheral clock genes that regulate the neurohumoral and cardiovascular pathways) and exogenous factors related to the sleep–wake behavioural pattern.

Based on the magnitude and direction of nocturnal BP change relative to daytime BP, four dipping profiles are recognized: (i) dippers (nocturnal BP fall between >10% and ≤20% of the daytime average BP); (ii) nondippers (nocturnal BP fall between ≥0% and ≤10% of the daytime average BP); (iii) reverse dippers or risers [nocturnal increase in systolic BP (SBP)]; and (iv) extreme dippers (nocturnal reduction of SBP >20%) [19]. Several other physiological and pathophysiological events including nighttime BP surges triggered by rapid-eye-movement sleep, nocturnal arousals, nocturia, obstructive sleep apnoea (OSA) episodes, as well as morning BP surge [18] further modulate this circadian rhythm of BP, leading to the different individual circadian variation of 24-h ambulatory BP (Fig. 1).

### Definition of nocturnal hypertension

**Figure 2:**
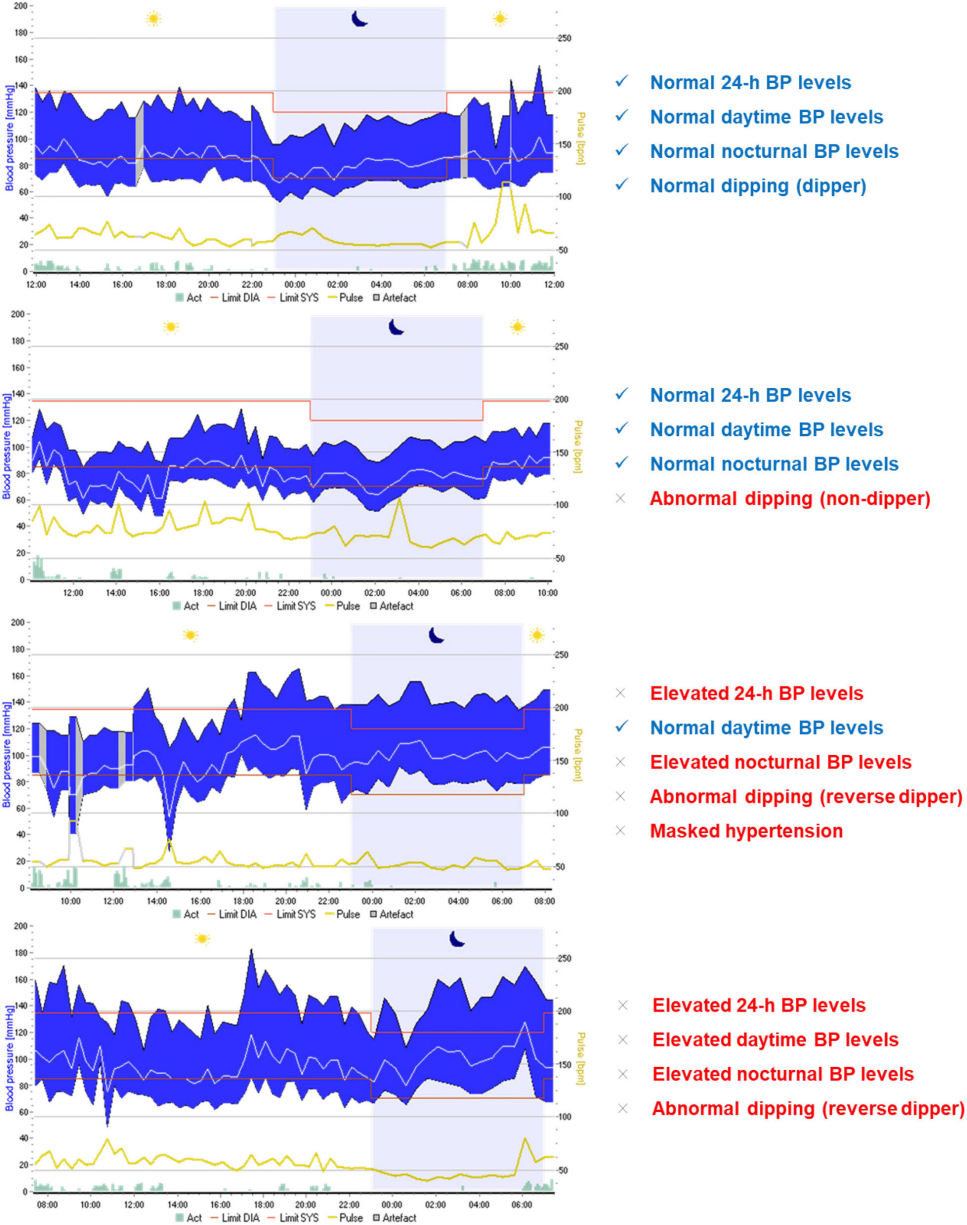
Examples of ambulatory BP phenotypes related to nocturnal hypertension and dipping status (individual patient data from the ABPM registry, 1st Department of Nephrology Aristotle University of Thessaloniki).

Different approaches have been proposed to describe nocturnal BP. The most common is to view it as a continuous variable and define normal versus elevated/hypertension in a categorical manner. To this end, the 2023 European Society of Hypertension Guidelines and the 2025 American Heart Association/American College of Cardiology (AHA/ACC) define nocturnal hypertension as an average nighttime SBP ≥120 (110 mmHg for the AHA/ACC) and/or diastolic BP (DBP) ≥70 (65 mmHg for the AHA/ACC) in an ambulatory BP measurement (ABPM), respectively [16]. Nocturnal hypertension may coexist with daytime hypertension or occur as isolated phenomenon (defined as isolated nocturnal hypertension), which is currently recognized as a distinct form of masked hypertension [19] (Fig. 2). Another way to define nocturnal hypertension is to describe it relative to daytime BP by quantifying the nocturnal BP decline (‘dipping’) and classifying individuals into the four aforementioned categories (dippers, nondippers, extreme dippers, and risers) (Figs. 1 and 2).

In addition to the above, the actual ‘nocturnal’ or ‘asleep’ period is defined in current literature using several approaches with varying precision. The definition may rely on fixed clock-time intervals, patient diaries, actigraphy, or polysomnography; while these approaches may affect individual dipping classification, their differential prognostic relevance has not been clearly established [19].

### EPIDEMIOLOGY **OF NOCTURNAL HYPERTENSION IN KTRs**

In both paediatric [20] and adult patients with kidney transplantation, the prevalence of nocturnal hypertension is particularly high, reaching up to 95% in some cohorts (Table 1). Of note, nocturnal hypertension is consistently more frequent than daytime hypertension; in an Italian cohort of 260 KTRs [21], the prevalence of nocturnal hypertension was >2-fold of that of daytime hypertension (74% vs. 30%). In another analysis of the same group in 170 KTRs, only 26% met hypertension criteria based on daytime thresholds (>135/85 mmHg), whereas 69% were classified as hypertensive using nighttime cutoffs (>120/70 mmHg) [22]. This predominance of elevated nighttime BP provides a key explanation for the very high rates of masked hypertension in the KTRs, ranging from 40% to 60% [23–26]. Importantly, in one of these studies [24], isolated nocturnal hypertension accounted for more than half of masked hypertension cases, highlighting nighttime BP abnormalities as a central driver of this phenotype.

**Table 1:** Main observational studies examining the prevalence of nocturnal hypertension and abnormal dipping patterns among KTRs.

Author (year)	Population	Nocturnal hypertension and abnormal dipping patterns definitions	Prevalence
Van den Dorpel *et al*. (1996) [39]	*N* = 18 adult KTRs >6 months post KTx	Nondipping: nocturnal MAP decline <10 mmHg	With CsA: 77.8%With AZA: 50%
Marx *et al*. (1996) [72]	*N* = 10 Kidney–pancreas Tx	Nondipping: <10% decline in nocturnal BP	Nocturnal increase in BP 80%
			100% nondippers
Kooman *et al*. (2001) [73]	*N* = 36 adult KTRs early post KTx	Nocturnal HTN: ≥125/75 mmHg in ABPM	Nocturnal HTN: 88.9%
			Nondippers: 94.5%
			Reverse dippers: 41.6%
		Nondipping: nocturnal MAP decline <10%	
Covic *et al*. (2003) [74]	*N* = 68 adult KTRs >12 months post KTx	Dipping: 1. 10 mmHg decrease of the daytime SBP; 2. 10% decrease in the daytime SBP; 3. night/day ratio <0.90 for SBP and <0.92 for DBP	1. 82.4%;2. 89.7%;3. 73.5%
Toprak *et al*. (2003) [51]	*N* = 35 adult KTRs ≥12 months post KTx	Nondipping: mean nocturnal MAP >10 than mean daytime MAP	60%
Oliveras *et al*. (2004) [75]	*N* = 23 adult KTRs >3 months post KTx	Nondipping: ≤10% reduction in MAP during sleep	Week 0 (without any antihypertensive treatment): 100% nondippersWeek 4 (after 4-week active treatment): 82.6% nondippers
Stenehjem *et al*. (2006) [76]	*N* = 49 adult KTRs >1 year post KTx	Nocturnal HTN: ≥120/70 mmHg in ABPM	Nocturnal HTN: 64%
		Nondipping: nocturnal SBP reduction ≥0 and ≤10%	Reverse dippers: 39% (SBP) and 28% (DBP)
		Reverse dipping: nocturnal SBP increase	
Wadei *et al*. (2007) [42]	*N* = 119 adult KTRs 1 year post KTx	Nondipping: ΔSBP >0 and <9%	Nondippers: 42%
		Reverse dipping: nocturnal rise in SBP	Reverse dippers: 34%
Sethna *et al*. (2009) [77]	*N*=33 paediatric KTRs >6 months post KTx	Nocturnal HTN: mean nocturnal BP >1 and mean nocturnal BP load >25%	Nocturnal HTN SBP > 1:13 (39%)
			Nocturnal HTN DBP > 1:12 (36%)
		Nondipping: <10% drop in mean BP	Nondipping: SBP 58%, DBP 42%
Steigerwalt *et al*. (2009) [78]	*N* = 38 adult KTRs ≥6 months post KTx SRL group: 18; CNI: 20	Nondipping: nocturnal MAP decline <10%	SRL group: 55.56% nondippersCNI group: 87.5% nondippers
Beltrán *et al*. (2010) [79]	*N* = 53 adult KTRs <10 years post KTx	Nondipping: nocturnal BP <10% lower than global mean for the diurnal BP/or nondipper in ABPM	Nondippers or risers: 45.3% of poorly controlled hypertensives (*n* = 4/28)
		Reverse dipping: global mean of nocturnal BP higher than global mean for diurnal BP	
Basiratnia *et al*. (2011) [50]	*N* = 66 KTRs aged 7–25 years >6 months post KTx	Nocturnal HTN: nocturnal BP ≥95th percentile	Isolated nocturnal hypertension: 25%
		Nondipping: <10% BP decline during nighttime	Nondipping:73%
Sezer *et al*. (2011) [47]	*N* = 82 adult KTRs ≥1 year post KTx	Nondipping: <10% nocturnal BP fall	65%
Ibernon *et al*. (2012) [44]	*N* = 126 adult KTRs 3 months post KTx	Nocturnal HTN: >120/75 mmHg in ABPM nondipping: 0 < ΔSBP < 10%/reverse dipping: nocturnal rise in SBP	Nondippers: 51.5%Reverse dippers: 31.1%
Azancot *et al*. (2014) [26]	*N* = 92 adult KTRs	Nocturnal HTN: ≥120/70 mmHg in ABPM dipping (ΔSBP ≥ 10%), nondipping (0 < ΔSBP < 10)	Nondippers: 79%Nocturnal HTN: 79%
Kayrak *et al*. (2014) [23]	*N* = 113 adult KTRs >12 months post KTx	Nondipping: decline <10% of daytime SBP	78%
Ahmed *et al*. (2015) [24]	*N* = 98 adult KTRs ≥1 year post KTx	Nocturnal HTN: >120/70 mmHg in ABPM	Isolated nocturnal hypertension: 33% (of masked)
		Nondipping: <10 mmHg drop of BP	Nocturnal HTN: 71%
Lee *et al*. (2015) [80]	*N* = 48 KTRs (1 year before and after KTx)	Nocturnal HTN: >120/75 mmHg in ABPM	Nondippers: 58 %
		Dipping (ΔSBP ≥ 10%), nondipping (ΔSBP between 0 and 9%), or reverse dipping (nocturnal rise in SBP).	
			Reverse dippers: 31 %
Mallamaci *et al*. (2016) [22]	*N* = 172 adult KTRs >1 year post KTx	Nocturnal HTN: nocturnal ABPM ≥120/70 mmHg	Nocturnal HTN: 69%
		Nondipping: night/day SBP ratio ≥1	Ratio ≥ 1:36%
Achim *et al*. (2016) [81]	*N* = 30 adult KTRs	Nondipping: nocturnal BP fall <10%	87.5%
Kolonko *et al*. (2018) [53]	*N* = 145 adult KTRs >1 year post KTx	dipping (ΔSBP ≥ 10%), nondipping (ΔSBP ≥0% and <10%), reverse dipping (with nocturnal SBP rise)	Reverse dippers: 12.5% (untreated hypertensives); 18.8% (treated with one drug); 16.2% (treated with two drugs); 27.3% (treated with three drugs); 38.5% (treated with four drugs)
Mallamaci *et al*. (2019) [21]	*N* = 260 adult KTRs >1 year post KTx	Nocturnal HTN: ≥120/70 mmHg in ABPM	Nocturnal HTN: 74%
		Nondipping: night/day SBP ratio ≥1	Nondippers: 33.1%
Mallamaci *et al*. (2020) [82]	*N* = 221 adult KTRs ≥4 months post KTx	Nocturnal HTN: ≥120/70 mmHg in ABPM	75.1%
Jaques *et al*. (2021) [45]	*N* = 123 adult KTRs. T0 discharge after KTx, T1: ABPM, T2: 1 year after ABPM, T3: 2 years after ABPM	Nondipping: nocturnal BP decline <10%	65.8%
Korogiannou *et al*. (2022) [10]	*N* = 204 adult KTRs ≥3 months post KTx	Extreme dipping (nocturnal BP fall >20%), dipping (fall >10% and ≤20%), nondipping (fall ≥0% and ≤10%), and reverse dipping (nocturnal increase in SBP).	Nondippers: 55.9%
			Reverse dippers: 29.4%
Eleftheriadis *et al*. (2024) [55]	*N* = 80 adult KTRs ≥6 months post KTx	Nocturnal HTN: ≥120/70 mmHg in ABPM;	Uncontrolled nocturnal: 93%
		Nondipping: ΔSBP 0%–9%;	Nondippers: 49%
		Reverse dipping: nocturnal increase in SBP	Reverse dippers: 40%

ABPM, ambulatory blood pressure monitoring; AZA, azathioprine; BP, blood pressure; CNI, calcineurin inhibitor; CsA, cyclosporine-A; DBP, diastolic blood pressure; HTN, hypertension; KTx, kidney transplantation; KTR, kidney transplant recipient; MAP, mean arterial pressure; SBP, systolic blood pressure; SRL, sirolimus.

Regarding dipping profiles, the majority of KTRs exhibit a blunted nocturnal dipping response. In an analysis from our group in 204 KTRs, absence of physiological nocturnal dipping was observed in 85% of patients, with 55% classified as nondippers and 30% as reverse dippers [10]. These findings are confirmed by a recent systematic review and meta-analysis, which reported a pooled prevalence of nondipping of 54% [95% confidence interval (CI) 45%–63%] [27]. However, substantial heterogeneity was noted across cohorts, with estimates ranging from as low as 8.1% in nondiabetic KTRs without left ventricular hypertrophy (LVH) to as high as 85% in hypertensive KTRs receiving tacrolimus.

#### Comparisons with predialysis CKD and dialysis patients

Both earlier [26] and more recent [28] comparative analyses with nondialysis chronic kidney disease (CKD) patients consistently identify elevated nocturnal BP as a hallmark feature of KTRs, despite a similar overall 24-h BP burden between the two populations. In a recent analysis from our group including 93 KTRs and 93 predialysis CKD patients matched for age, sex, and estimated glomerular filtration rate (eGFR), nighttime DBP was significantly higher in KTRs, whereas daytime and 24-h BP values were comparable between groups [28]. Importantly, this nocturnal BP phenotype in KTRs is accompanied by a higher prevalence of abnormal dipping profiles; in a meta-analysis including 788 patients, KTRs had a significantly higher likelihood of exhibiting a nondipper pattern compared with their CKD counterparts (Odds ratio 1.79, 95% CI 1.01–3.20) [29].

With regards to comparisons with dialysis populations, the available evidence is less consistent, although it similarly underscores the elevated nocturnal BP after kidney transplantation. In a 2014 analysis, KTRs exhibited significantly higher nocturnal BP levels at both 3 and 12 months after transplantation compared with patients undergoing either haemodialysis or peritoneal dialysis [26]. In contrast, a more recent study reported significantly lower nocturnal BP in KTRs compared with both the first and second 24-h periods of the interdialytic interval in haemodialysis patients despite persistence of nondipping profiles [10]; nevertheless, in both groups the mean nocturnal BP levels were overall high, highlighting the persistently high burden of nocturnal hypertension in both dialysis and KTRs.

### PATHOPHYSIOLOGY **OF NOCTURNAL HYPERTENSION IN KTRs**

The mechanisms underlying nocturnal hypertension and abnormal dipping patterns in kidney transplantation are not yet fully elucidated. Current evidence suggests combined influence of persistent CKD-related pathophysiology and transplant-specific factors, including immunosuppressive therapy and progressive allograft dysfunction.

#### CKD-related factors

##### Sodium and water homeostasis

Circadian BP variation is modulated by overall kidney function and renal sodium handling. Impaired sodium excretion coupled with increased sodium intake is suggested to be the major determinant of the development of nocturnal hypertension and nondipping BP patterns, and conversely sodium restriction, diuretics, and aldosterone antagonists can decrease nocturnal BP and possibly restore nocturnal BP fall [15].

From a pathophysiological perspective, these phenomena can be explained by the circadian alterations in renal sodium excretion regulated through the ‘pressure–natriuresis’ mechanism [30]. In settings of reduced eGFR, and/or enhanced tubular sodium reabsorption (i.e. in patients with sodium-sensitive phenotypes) daytime sodium retention may occur. As a compensatory response, BP increases during nighttime to excrete the sodium excess through pressure–natriuresis mechanism and thereby maintain balance [30]. This nocturnal natriuretic shift commonly also increases episodes of nocturia, a common clinical feature in CKD, further impairing sleep quality and amplifying sympathetic activation, thereby establishing a vicious cycle contributing to nocturnal BP elevation [31]. In the context of overall increased dietary sodium intake and particularly in cases of increased sodium intake in the evening and night hours (late dinners, salty snacks, etc.), these phenomena further exaggerate.

##### Renin–angiotensin system

As eGFR declines, there is often an inappropriate activation of the renin–angiotensin system (RAS) which may blunt the physiological nocturnal BP decline through salt retention and vasoconstriction, as well as through interactions with the sympathetic nervous system (SNS) [14]. Moreover, chronic elevation of angiotensin II levels has been associated with endothelial dysfunction and increased vascular stiffness, that are also known to be associated with higher BP levels during nighttime in CKD [14, 32]. Although systemic RAS is not overly activated in KTRs, this may not reflect the intrarenal RAS activity [33] creating a plausible pathophysiological link between the dysregulation of the renin–angiotensin axis and circadian BP rhythm disruption in KTRs, consistent with observations in CKD.

##### Autonomic nervous system and other factors

Sleep-related changes in BP are closely linked to autonomic regulation. Increased SNS activity is a well-established contributor to nocturnal hypertension, particularly in CKD, where sympathetic drive is chronically elevated [34]. In kidney transplantation, sympathetic overactivation may persist early after surgery due to signalling from the native kidneys, while the graft itself is initially denervated and only gradually undergoes partial reinnervation over time [35]. Furthermore, disruption of circadian rhythm regulated by the suprachiasmatic nucleus of the hypothalamus mediated via upregulation of angiotensin-I or uremic state, and impaired sleeping pattern (including later onset, shorter duration, and increased fragmentation of sleep cycle) are also considered to play a role [14, 29, 36, 37].

#### Transplant-specific factors

##### Immunosuppression

Immunosuppressive regimens, mainly corticosteroids and calcineurin inhibitors (CNIs) may also be contributing factors to nocturnal hypertension. Corticosteroids promote extracellular volume expansion through direct sodium retention, increase insulin resistance and hyperinsulinemia that further impair normal natriuresis [38], and heighten vascular reactivity. CNIs in turn, induce afferent arteriolar vasoconstriction, impair nitric oxide bioavailability, and enhance distal tubular sodium and water reabsorption, thereby favouring sodium sensitivity [36]. In a preliminary mechanistic study [41], exposure to cyclosporin-A was associated with higher nocturnal BP and more impaired dipping compared to azathioprine. Post-transplant diabetes development through the above and other mechanisms further promotes nighttime BP increase and abnormal dipping [41].

##### Recipient and donor phenotypes

Several recipient characteristics have been associated with abnormal nocturnal BP patterns. Nondipping and reverse dipping profiles are reported more frequently in older recipients [42], males [43], diabetics [23, 44], and those with high body mass index [44]. Donor-related factors may also contribute, as recipients of deceased-donor grafts have been reported to exhibit higher nocturnal SBP levels compared with recipients of living-donor transplants [23], potentially reflecting differences in ischemia–reperfusion injury or baseline graft function.

##### Graft dysfunction

Impaired graft function also appears to be closely associated with higher incidence of nocturnal hypertension and abnormal dipping profiles after transplantation surgery, similarly to the relationship of reduced eGFR and nocturnal hypertension in nontransplant populations [15]. A meta-analysis pooling seven observational studies (*n* = 788) demonstrated that nondipping and reverse dipping patterns were more frequent in KTRs with impaired graft function, as evidenced by a significantly higher eGFR in dippers compared to nondippers and reverse dippers [29]. The group of reverse dippers showed the worst kidney function, suggesting a graded relationship between dipping status and graft function. Moreover, no significant differences were observed between dippers and nondippers in the risk of acute rejection episodes, renal resistive index (RRI), lipid profile, and 24-h proteinuria, indicating that abnormal nocturnal BP patterns are more closely linked to chronic haemodynamic alterations and long-term graft dysfunction rather than acute events or metabolic disturbances.

### PROGNOSTIC **SIGNIFICANCE OF NOCTURNAL HYPERTENSION IN KTRs**

To this date, several observational studies have evaluated the association of nocturnal BP elevation and abnormal dipping profiles with adverse graft (Table 2) and cardiovascular outcomes (Table 3) in KTRs.

**Table 2:** Main observational studies examining the associations of nocturnal hypertension and abnormal dipping patterns with adverse graft outcomes in KTRs.

Author (year)	Population	Study design	Predictor variable	Endpoints	Results
Wadei *et al*. (2011) [42]	*N* = 36 KTRs without rejection history ≥1 year post KTx	Prospective cohort	Dipping status	eGFR, histological findings in kidney transplant biopsy	Compared with dippers, reverse, and nondippers had: higher Banff cv score at 1 year (*P* = .03); lower eGFR at last follow-up (73.7 ± 18.1, 55.7 ± 16.3, and 56.6±21 mL/min/1.73 m^2^ for dippers, non-, and reverse dippers, respectively, *P* = .05); higher kidney function loss (8.0 ± 20, −9 ± 17, and 1 ± 14 mL/min/1.73 m^2^ for dippers, non-, and reverse dippers, respectively, *P* = .02); eGFR at 4 years and at last follow-up independently correlated with ΔSBP at 1 year (*r* = 0.46, *P* = .01; *r* = 0.34, *P* = .03).
Sezer *et al*. (2011) [47]	*N* = 82 adult KTRs ≥1 year post KTx	Cross-sectional	Dipping vs nondipping status	RRI ≥ 0.7	Incidence of an increased RRI was higher in nondippers vs. dippers (52% vs. 24%; *P* = .01)Predictive factor associated with nondipper status: RR: 2.2 (1.16, 4.42)Independent factor associated with dipping vs. nondipping status (R^2^ = 0.053; *P =* .05)
Ibernon *et al*. (2012) [44]	*N* = 126 adult KTRs 3 months post KTx	Prospective cohort	Reverse dipper status at 3 months	CV events (MI, angina, ischaemic stroke, transient cerebral ischaemic attack, congestive heart failure, and aortic aneurysm rupture)Combined outcome: CV events or graft failure for any reason	For CV events: RR: 3.65, 95% CI (1.15–11.5)For combined outcome: Univariate: RR 3.38, 95%CI (1.45–7.87), *P* = .004 Multivariate: RR 3.50, 95% CI (1.36–8.93), *P* = .009
Mallamaci *et al*. (2018) [46]	*N* = 260 adult KTRs ≥1 year post KTx	Prospective cohort	Nighttime SBP	Combined kidney endpoint: eGFR loss >30%, end-stage kidney disease or death	Combined endpoint: nighttime SBP (per 5 mmHg increase):adjusted HR 1.10, 95% CI (1.03–1.17)eGFR trajectories: nighttime SBP (per 5 mmHg increase):adjusted −1.07 ml/min/1.73 m^2^ (−1.23, −0.90)
Jaques *et al*. (2021) [45]	*N* = 123 adult KTRs; T0 discharge after KTx, T1: ABPM, T2: 1 year after ABPM, T3: 2 years after ABPM	Prospective cohort	Systolic dipping status	eGFR	In multivariate analysis, systolic dipping status was positively associated with eGFR (*P* = .009).Dippers had a 10.4 mL/min/1.73 m^2^ higher eGFR compared to nondippers.
Gurlek *et al*. (2026) [40]	*N* = 74 KTRs with mean time post KTx 34.9 ± 1.7 months	Cross-sectional	Nocturnal DBP	RRI (group 1 RRI < 0.67; *n* = 29 and group 2 RRI ≥ 0.67; *n* = 45); LVMI	Nocturnal DBP was positively correlated with RRI (*P* = .01) and LVMI (*P* = .033). Group 1 had lower nocturnal DBP (*P* = .01).In multiple regression analysis RRI (*P* = .012) and LVMI (*P* = .02) were predictors of nocturnal DBP.

ABPM, ambulatory blood pressure monitoring; CI, confidence interval; CV, cardiovascular; DBP, diastolic blood pressure; eGFR, estimated glomerular filtration rate; HR, hazard ratio; KTx, kidney transplantation; KTR, kidney transplant recipient; LVMI, left ventricular mass index; MI, myocardial infarction; RR, relative risk; RRI, renal resistive index; SBP, systolic blood pressure.

**Table 3:** Main observational studies examining the associations of nocturnal hypertension and abnormal dipping patterns with adverse cardiovascular outcomes in KTRs.

Author (year)	Population	Study design	Predictor Variable	Endpoints	Results
Lipkin *et al*. (1993) [40]	*N* = 28 adult KTRs ≥1 year post KTx	Cross-sectional	Nocturnal SBP, dipping status	LVMI	Significantly higher LVMI in nondippers vs. dippers (124 ± 15 vs. 89 ± 7 g/m^2^, *P* < .05)
					Nocturnal SBP was positively correlated with LVMI (*r* = 0.68, *P* < .001)
Toprak *et al*. (2003) [51]	*N* = 35 adult KTRs ≥12 months post KTx	Cross-sectional	Nocturnal BP, dipping status	LVMI	Significantly higher LVMI in the nondipping group (133 ± 35 vs. 109 ± 26 g/m^2^, *P* = .04)
					On stepwise multiple regression analysis, nighttime SBP load and Hb were independent predictors of LVMI (*r*^2^ = 0.53)
Basiratnia *et al*. (2011) [50]	*N* = 66 KTRs aged 7–25 years >6 months post KTx	Cross-sectional	Nocturnal BP, dipping status	LVMI	Significant association between all ABPM parameters and LVMI (*r* = 0.28–0.38, *P* = .025–.007)
Ibernon *et al*. (2012) [44]	*N* = 126 adult KTRs 3 months post KTx	Prospective cohort	Reverse dipper status at 3 months	CV events (MI, angina, ischaemic stroke, transient cerebral ischaemic attack, congestive heart failure, and aortic aneurysm rupture) Combined outcome: CV events or graft failure for any reason	For CV events: RR 3.65, 95% CI (1.15–11.5), *P* = .0276For combined outcome: Univariate: RR 3.38, 95% CI (1.45–7.87), *P* = .004 Multivariate: RR 3.50, 95% CI (1.36–8.93), *P* = .009
Sezer *et al*. (2013) [48]	*N* = 98 adult KTRs ≥1 year post KTx	Cross-sectional	Nocturnal SBP	LVMI	LVMI positively correlated with mean nighttime SBP/DBP (*r* = 0.312/0.427, *P* = .007/.005, respectively)
					Mean and max nighttime SBP and RRI were independent risk factors for LVMI (*P* = .001/.035/.05, respectively)
Ozkayar *et al*. (2014) [52]	*N* = 73 adult KTRs ≥3months post KTx	Cross-sectional	Nocturnal SBP	FMD ≤ 10%	In KTRs with endothelial dysfunction, nocturnal SBP was higher (126 ± 16 vs. 117 ± 12 mmHg, *P* = .029)
Mallamaci *et al*. (2016) [22]	*N* = 172 adult KTRs >1 year post KTx	Cross-sectional	Nocturnal SBP, nighttime/daytime SBP ratio	cIMT	Average nighttime SBP (*r* = 0.24, *P* = .001), night/day SBP ratio (*r* = 0.23, *P* = .002), and 24-h SBP (*r* = 0.16, *P* = .04) were positively correlated to cIMT
					Adjusted multiple regression analysis night/day SBP ratio showed independent association with cIMT (β = 0.14, *P* = .04)
Kolonko *et al*. (2018) [53]	*N* = 145 adult KTRs >1 year post KTx	Cross-sectional	Nocturnal SBP dipping status	PWV	Nocturnal SBP dipping status was positively correlated with PWV (*r* = 0.154, *P* = .06)

ABPM, ambulatory blood pressure monitoring; CI, confidence interval; CV, cardiovascular; cIMT, carotid intima-media thickness; DBP, diastolic blood pressure; Hb, haemoglobin; KTx, kidney transplantation; KTR, kidney transplant recipient; LVMI, left ventricular mass index; MI, myocardial infarction; PWV, pulse wave velocity; RR, relative risk; RRI, renal resistive index; SBP, systolic blood pressure; FMD, flow-mediated dilatation.

**Figure 3:**
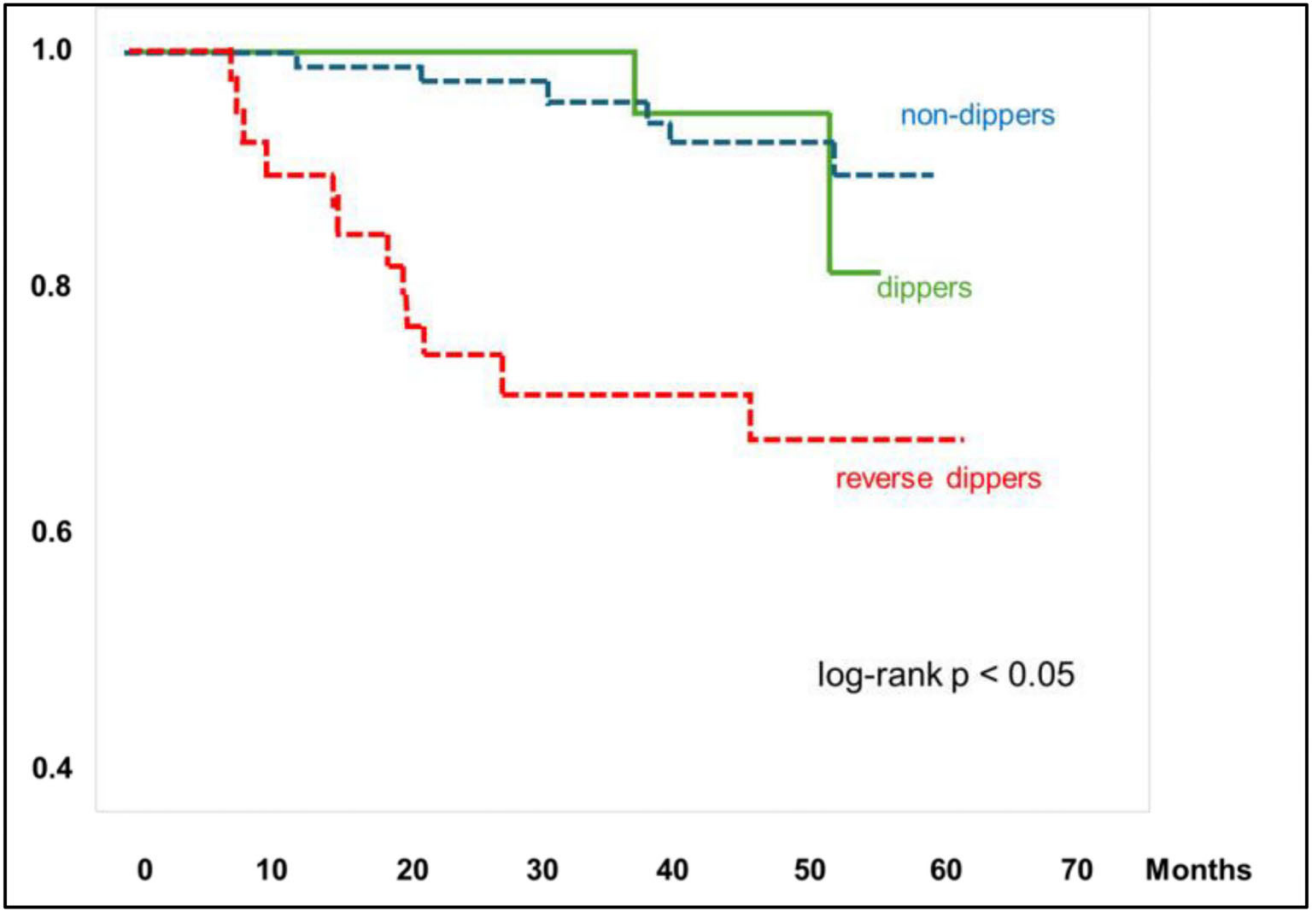
Cumulative survival for the combined endpoint of cardiovascular event or graft failure according to dipping status. Redrawn and adapted from the results reported by Ibernon *et al*. [44].

Early evidence supporting the prognostic relevance of circadian BP disruption in KTRs was provided by Ibernon *et al*. in 126 KTRs that underwent ABPM at 3 months post-transplantation; an adverse dipping pattern was the only BP-derived parameter independently associated with the combined endpoint of graft failure or cardiovascular events during mean follow-up of 45 months (Fig. 3) [44]. A smaller study of 36 KTRs with ABPM 1 year after transplantation, showed that reverse dippers and nondippers exhibited more advanced vascular lesions on protocol biopsy (higher Banff cv scores), lower eGFR, and greater functional decline during follow-up of up to 4 years compared with dippers [42]. Similarly, Jaques *et al*. reported that SBP dipping was positively associated with eGFR in longitudinal regression models up to 2 years after ABPM assessment [45].

Beyond categorical dipping patterns, nocturnal BP levels per se are also independent predictors of adverse graft outcomes. In the largest longitudinal cohort study (260 KTRs) [46], Mallamaci *et al*. showed that higher nighttime SBP was independently associated with an increased risk for the combined kidney endpoint of >30% eGFR decline, progression to end-stage kidney disease, or death over a median follow-up of 3.7 years (hazard ratio 1.10, 95% CI 1.03–1.17 per 5 mmHg increase). Additional studies have linked nocturnal hypertension to intermediate markers of graft injury. Sezer *et al*. observed that nondipping recipients exhibited more frequently an elevated RRI (≥0.7) [47], suggesting impaired intrarenal haemodynamics, while subsequent analyses showed strong associations between nighttime BP and RRI [48, 49].

In parallel, multiple studies have demonstrated associations between nocturnal BP abnormalities and cardiovascular damage in KTRs. In the aforementioned prospective study of Ibernon *et al*., a reverse dipping pattern was associated with a 3.6 higher risk of cardiovascular events (myocardial infarction, angina, ischaemic stroke, transient cerebral ischaemic attack, congestive heart failure, aortic aneurysm rupture) (Fig. 3) [44]. Beyond hard clinical outcomes, both early and more contemporary reports, in children [50] and adult [40, 48, 50, 51] KTRs, have consistently linked higher nighttime SBP to adverse cardiac remodelling. Several studies demonstrated associations between nocturnal SBP and left ventricular mass index, while nondipping or reverse dipping profiles were frequently linked with the presence of LVH [40, 48, 50, 51]. Moreover, accumulating evidence suggests that nocturnal hypertension may contribute to subclinical vascular injury in KTRs. In 172 recipients, nighttime SBP and the night-to-day BP ratio (but not daytime or office BP) were the only parameters independently associated to carotid intima-media thickness [22]. Consistently, KTRs with higher nocturnal SBP have been reported to have impaired endothelial function assessed by flow-mediated dilatation (FMD) [52], as well as higher arterial stiffness [53].

### DIAGNOSIS **OF NOCTURNAL HYPERTENSION IN KTRs**

ABPM represents the gold standard for the diagnosis of nocturnal hypertension, as it enables comprehensive quantification of nocturnal BP load, characterization of dipping status, and evaluation of the morning BP surge and short-term BP variability [19]. Beyond its critical role in assessment of circadian BP variations, ABPM provides objective 24-h BP profiling under real-life conditions, allowing assessment of BP behaviour during usual daily activities, and shows superior predictive value for target-organ damage and hard cardiovascular outcomes compared with office BP [19, 54]. Importantly, ABPM is the gold standard for identifying masked and white-coat hypertension, phenotypes that are also associated with adverse outcomes [19, 54]. These advantages are particularly relevant in KTRs, given the high frequency of masked hypertension in this population [36]. Of note, agreement between office BP and 24-h ambulatory, daytime, and nighttime BP is poor, with the weakest correlation observed between office and nighttime BP [21, 24].

In the last years, nocturnal home BP monitoring has been explored as an additional approach for nocturnal BP assessment in the general hypertensive population [15]; preliminary evidence suggests acceptable agreement with ABPM, both in terms of absolute BP values and detection of abnormal dipping profiles [54]. In KTRs, data validating nocturnal home BP monitoring is lacking. Indirect evidence deriving from a recent analysis with conventional home BP measurements, office BP, and 24-h ABPM showed that nearly half (46%) of recipients with ABPM-defined nocturnal hypertension were misclassified as normotensive when relying on home measurements alone [55]. Thus, to date, ABPM remains indispensable and the only established method for the accurate diagnosis of nocturnal hypertension and the reliable characterization of dipping profiles after transplantation.

### MANAGEMENT **OF NOCTURNAL HYPERTENSION IN KTRs**

**Figure 4:**
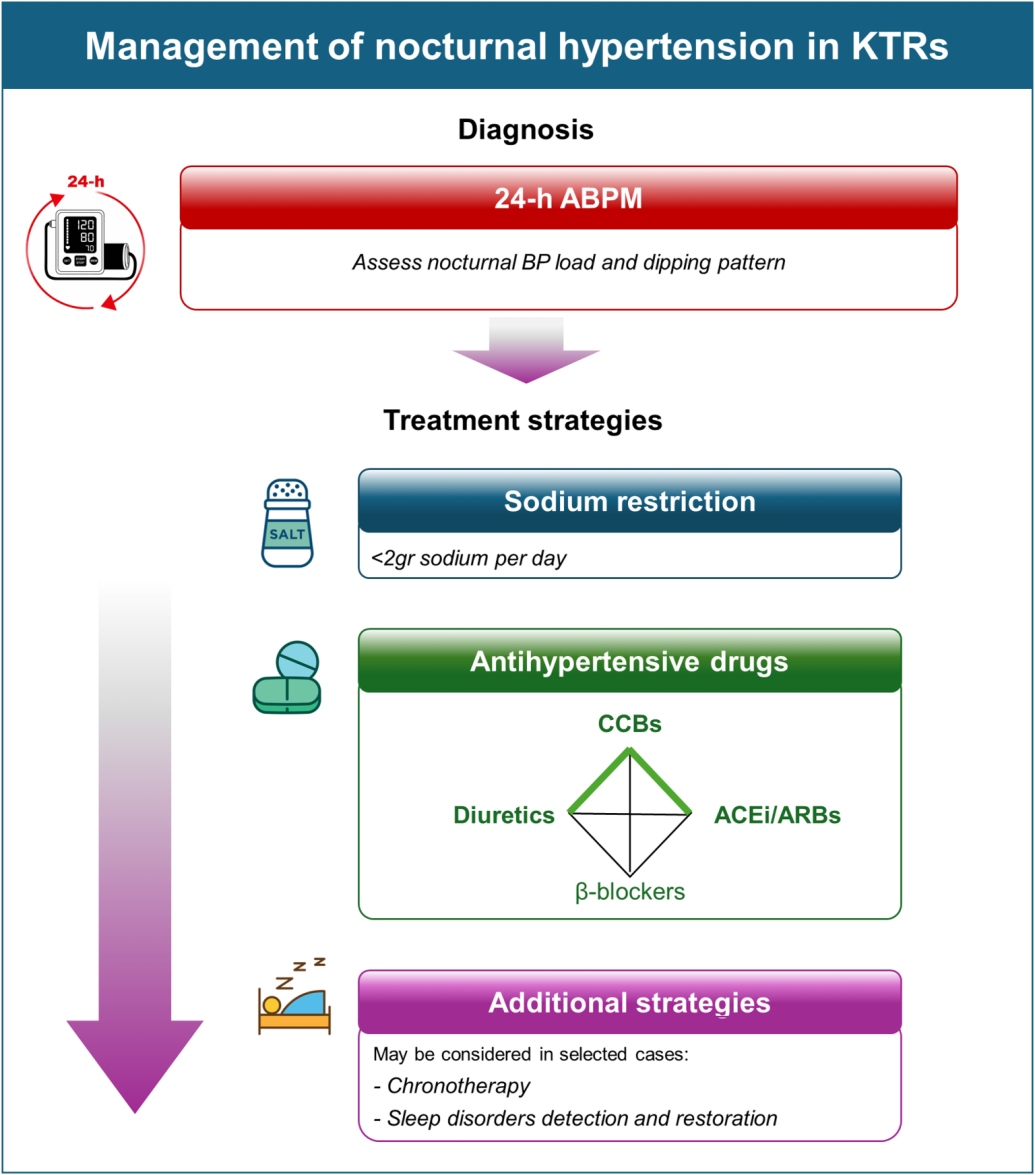
Algorithm for diagnosis and treatment of nocturnal hypertension in KTRs.

Specific evidence to guide the therapeutic management of nocturnal hypertension and abnormal dipping status in KTRs is absent, and most approaches are extrapolated from the general and CKD populations. As in other high-risk groups, the primary goal is sustained 24-h BP control, with particular emphasis on reducing nocturnal BP load [15], with strategies such as those summarized in Table 4 and in the algorithm in Fig. 4. Of importance, it should not be forgotten that pretransplant hypotension has also been associated with delayed graft function and reduced graft survival [56], underscoring the bidirectional relationship between BP and graft outcomes. Although there are no detailed data on the associations of hypotension over mid-term and long-term periods post renal transplantation with outcomes, one must be careful not to overtreat patients. Further, there are no randomized studies examining the optimal office, home, or ambulatory BP in KTRs; so, the physicians need to follow the guidance for this population extrapolated for studies in CKD, recommending an office target <130/80 mmHg [16, 57].

**Table 4:** Main strategies suggested for treatment of nocturnal hypertension and abnormal dipping status in KTRs.

Intervention	Involved mechanism	Evidence
**Sodium restriction and optimization of volume status**	Preventing sodium retention and excess nighttime natriuresis	No randomized evidence available. Indirect evidence in KTRs: association of 24-h sodium excretion and BP [59–61]; short-term dietary sodium restriction (<80 mmol/day) reduces 24-h and nocturnal BP [62].
**Antihypertensive medication**		No randomized evidence specifically targeting nocturnal hypertension is available and no dedicated recommendations exist.
		Management should focus on achieving sustained 24-h BP control, with antihypertensive therapy selected according to hypertension guidelines for KTRs.
CCBs	Dihydropyridine CCBs additionally counteract the preglomerular vasoconstrictive effects of CNIs	First line agents for hypertension; long-acting dihydropyridine CCBs associated with favourable effects on kidney function and long-term graft outcomes in KTRs [36, 37, 64, 65, 83].
Diuretics	Reduction of sodium and volume overload; thiazide/thiazidelike diuretics additionally counteract CNI-induced sodium retention	First line agents for hypertension. Indirect pathophysiological data from CKD patients: advanced CKD is commonly associated with nondipping and reverse dipping phenotypes and diuretics or salt restriction may restore normal dipping [14, 58, 65].
ACEIs/ARBs	RAS blockade with additional effects on vascular function and sympathetic activity	First line agents for hypertension, favourable effects on albuminuria reduction; preferably started after achieving stable graft function [36, 37, 64, 65, 83].
β-blockers	Target SNS overactivity	Add-on therapy, may be considered in the presence of sympathetic overactivity [29].
**Chronotherapy**	Evening or bedtime dosing of antihypertensives targeting nighttime BP with consistent 24-h BP control	No randomized evidence available. Indirect data: evening dosing lowers nighttime BP [66], but no cardiovascular benefits [67, 68]. A single noncontrolled trial in CKD: evening dosing lowered nocturnal BP and restored normal circadian rhythm in 87.5% of patients [69].
**Sleep disorders detection and restoration**	Sleep disorders (mainly OSA) restoration attenuates intermittent hypoxia and reduces sympathetic overactivity	No randomized evidence available. Indirect evidence from general population: management of OSA (weight reduction, CPAP, etc.) achieve modest but clinically meaningful BP reductions, more pronounced for nocturnal BP [71].

Abbreviations: ACEIs/ARBs, angiotensin-converting-enzyme inhibitor/angiotensin-receptor blocker; BP; blood pressure; CCB, calcium channel blocker; CNI, calcineurin inhibitor; CKD, chronic kidney disease; CPAP, continuous positive airway pressure; OSA, obstructive sleep apnoea; KTR, kidney transplant recipient; SNS, sympathetic nervous system; RAS, renin–angiotensin system.

Dietary sodium restriction together with optimization of volume status, should be central strategies, as these interventions may help restore normal dipping pattern by preventing sodium retention and excess nighttime natriuresis [58]. Pilot studies have confirmed the relationship between 24-h urinary sodium excretion and BP levels in KTRs [59–61], while in a study with 38 hypertensive KTRs, short-term dietary sodium restriction (<80 mmol/day) for a 14-day period has been shown to lead in significant reductions in 24-h ambulatory and nocturnal BP levels [62].

With regards to antihypertensive therapy, current guidelines do not recommend specific agents for nocturnal hypertension either in the general CKD or in KTRs due to limited long-term outcome evidence [16]. Nonetheless, long-acting dihydropyridine calcium channel blockers are commonly recommended in KTRs based on favourable effects on kidney function and long-term graft outcomes [16, 36, 37, 63, 64]. Angiotensin-converting-enzyme inhibitors and angiotensin-receptor blockers are recommended in patients with albuminuria [16, 36, 37, 63, 64] and may be helpful in nocturnal hypertension due to effects in vascular function and sympathetic activity. Thiazide/thiazidelike diuretics may provide additional benefit, including counteracting cyclosporine-mediated sodium retention [65], while β-/α-blockers may be considered in the presence of sympathetic overactivity [29].

In the general hypertensive population, chronotherapy remains controversial. Meta-analyses indicate potential reductions in nighttime BP with bedtime dosing [66], but methodological concerns persist, and large pragmatic trials have not demonstrated cardiovascular benefits of evening versus morning administration [67, 68]. Accordingly, current recommendations prioritize long-acting agents that ensure consistent 24-h BP control [15–17]. No direct evidence exists on chronotherapy in KTRs, and only limited data are available in nondialysis CKD. A previous study in 32 nondipper CKD patients showed that shifting one antihypertensive drug from morning to evening dosing decreased nocturnal BP and restored dipping status to normal (87.5% dippers after intervention) [69]. As such, individualized evening dosing strategies may be considered in selected patients with confirmed isolated nocturnal hypertension, until further evidence is available.

Finally, sleep disorders, particularly OSA, are common in CKD and KTRs and are strongly associated with nocturnal hypertension and nondipping patterns [15, 70]. Management includes weight reduction, continuous positive airway pressure, and mandibular advancement devices. Meta-analyses suggest these interventions achieve modest but clinically meaningful BP reductions, often more pronounced for nocturnal than office BP, supporting further systematic examination of sleep-disordered breathing in KTRs with nocturnal hypertension [71].

### CONCLUSIONS **AND FUTURE RESEARCH NEEDS**

Nocturnal hypertension and abnormal dipping profiles are highly prevalent in KTRs and represent a dominant yet universally under-recognized phenotype of post-transplant hypertension. These disturbances arise from the interplay between persistent CKD-related mechanisms (i.e. impaired sodium handling, neurohumoral activation, and autonomic dysfunction) and transplant-specific factors including immunosuppressive therapy and progressive allograft dysfunction. Abundant evidence consistently links nocturnal BP elevation and nondipping patterns with adverse graft outcomes and increased cardiovascular risk, and this association is stronger compared to that of daytime BP.

Based on the above, a paradigm shift in post-transplant BP management is needed. Reliance on office BP readings alone is insufficient, and broader implementation of ABPM should be prioritized to detect masked and isolated nocturnal hypertension phenotypes and to guide individualized management (Fig. 4); nocturnal BP should be increasingly recognized as a clinically meaningful and potentially modifiable therapeutic target in transplantation. Future research priorities should include dedicated transplant-specific studies to better characterize the epidemiology and mechanisms of circadian BP disruption, evaluating the impact of different immunosuppressive strategies on nocturnal BP (including potential effects of steroid-sparing and CNI-sparing schemas), and define optimal treatment approaches for patients with isolated nocturnal hypertension. Ultimately, randomized trials are needed to determine whether interventions specifically targeting nocturnal hypertension translate into better nighttime BP, restoration of dipping profiles, and improved cardiovascular and graft outcomes in KTRs. Until such evidence becomes available, use of ABPM to assess nocturnal BP and circadian BP phenotypes in routine follow-up represents an actionable opportunity to address residual cardiovascular risk and improve long-term prognosis after kidney transplantation.

## Data Availability

No new data were generated or analysed in support of this research.
